# Bayesian Modeling of Bovine Tuberculosis Prevalence for Enhanced Estimation and Control in Intensive Dairy Farms: A Longitudinal Decision‐Support Tools for Veterinarians and Policymakers

**DOI:** 10.1155/vmi/2737085

**Published:** 2026-09-23

**Authors:** Berhanu Abera, Balako Gumi, Gobena Ameni, Rebecca L. Smith, Gezahegne Mamo

**Affiliations:** ^1^ Ethiopian Institute of Agricultural Research, Addis Ababa, Ethiopia, eiar.gov; ^2^ College of Veterinary Medicine and Agriculture, Addis Ababa University, P.O. Box 34, Bishoftu, Ethiopia, aau.edu.et; ^3^ Aklilu Lemma Institute of Pathobiology, Addis Ababa University, P.O. Box: 1176, Addis Ababa, Ethiopia, aau.edu.et; ^4^ Department of Veterinary Medicine, College of Agriculture and Veterinary Medicine, United Arab Emirates University, P.O. Box 15551, Al Ain, United Arab Emirates, uaeu.ac.ae; ^5^ Department of Pathobiology, College of Veterinary Medicine, University of Illinois, Urbana, Illinois, USA, illinois.edu

**Keywords:** apparent prevalence, Bayesian modeling, bovine tuberculosis, disease estimate, true prevalence

## Abstract

Accurate prevalence estimation is crucial for effective bovine tuberculosis (bTB) control, as unreliable data often gives rise to strategic failures, misallocation of resources, and loss of stakeholder trust. In an epidemiological study, obtaining true prevalence (Tp) requires accounting for misclassification and measurement errors inherent in diagnostic testing. Given the imperfect specificity and sensitivity of single intradermal comparative tuberculin tests (SICCTT), Bayesian methods present a natural way to propagate these uncertainties. This study uses a Bayesian approach to improve bTB prevalence estimates from imperfect diagnostic tests. It transforms raw screening data from a dairy farm case study into reliable evidence for better veterinary decisions and targeted disease control policies. Prior sensitivity estimates of SICCTT under standard and severe interpretations were 70.0% (BPI: 52.0%–84.6%) and 78.68% (BPI: 62.1%–90.5%), with corresponding specificities of 96.4% (BPI: 92.2%–98.8%) and 94.7% (BPI: 91.4%–97.1%). Posterior estimates showed a slight reduction in sensitivity under standard interpretation (68.3%, BPI: 50.4–83.7), while other estimates remained comparable to prior distributions. Bayesian modeling revealed notable variation in Tp across testing rounds. Rounds 1 and 3 had the highest prevalence, 27.6% (BPI: 18.5–40.6) and 30.9% (BPI: 23.3–47.5) under standard interpretation, respectively. In contrast, test round 4 yielded the lowest prevalence estimate at 4.1% (BPI: 0.6–8.6). The posterior probability of Tp being below 1%, the threshold for herd freedom from *M. bovis* infection, was 0% for the first three test rounds, but increased to 5.8% in test round 4. Comparative analysis of estimation methods showed that Bayesian estimators with informative priors produced slightly higher point estimates for Tp than the Rogan–Gladen estimator and Bayesian models with uninformative priors, except in test round 3. However, Bayesian estimation with informative priors exhibited wider credible intervals and strong coverage, reflecting the added uncertainty from not fixing test sensitivity and specificity. Convergence diagnostics, including trace plots, autocorrelation function (ACF) plots, and Gelman–Rubin statistics, confirmed adequate mixing and stationarity of the Markov chains, indicating satisfactory performance of the Markov Chain Monte Carlo (MCMC) algorithms. Rapid autocorrelation decay and the stabilization of Gelman–Rubin statistics near 1.0 provide evidence that the posterior samples adequately represent the target distributions. In conclusion, classic apparent prevalence estimates are overly precise when uncertainty around test performance is high. These Bayesian approaches provide a more accurate estimate of bTB prevalence in the study herd and provide baseline data for future Tp estimates using linked combined data.

## 1. Introduction

Ethiopia’s rapidly growing dairy sector struggles with increased incidence of bovine tuberculosis (bTB) due to intensive animal husbandry and periurban livestock systems [1].

A typical strategy for bTB control in domesticated animals involves regular field tests and quarantine of infected herds. This prevents disease from spreading beyond the herd, while slaughter of diseased animals removes the infection from the herd [2]. However, the true disease state is rarely known in practice, due to inherent limitations of bTB tests [3].

In Ethiopia, the single intradermal comparative cervical tuberculin test (SICCTT) is the most commonly applied ante‐mortem bTB diagnostic tool in cattle, as documented in several epidemiological studies [4–7]. Unfortunately, it has its own constraints, missing some infected animals from culling or leading to unnecessary culling [3, 8–10]. Those undetected infected animals may remain infectious and play a great role in disease transmission.

Accurate prevalence estimation is a critical need for effective bTB control. Without reliable prevalence data, control programs lack actionable intelligence, leaving interventions vulnerable to strategic failure, resource misallocation, and eroded stakeholder confidence [11, 12]. An inaccurate prevalence estimate is not a neutral error; it actively sabotages control programs by furnishing false intelligence [13]. Without knowing the true extent and distribution of the disease, interventions devolve into guesswork, leading to one of two costly failures. The overkill wastes limited resources on draconian measures in disease‐free areas, eroding farmer trust and provoking resistance to control efforts [14]. The second, under‐reaction, allows silent epidemics in high‐risk populations to propagate unchecked, leading to endemic disease and exponentially increasing the cost and difficulty of future eradication [15–17].

Prevalence estimation in epidemiological studies often relies on diagnostic tests to classify disease status with respect to the binary trait [18]. In the absence of a gold standard and without external constraints, parameter estimation assumes constant sensitivity/specificity and conditional independence of tests, but these assumptions may require restrictions due to being criticized as unrealistic [19]. Estimating the true prevalence (Tp) thus becomes a matter of adding constraints on the parameters. These constraints must come from external sources, such as prior studies or expert opinion for example from prior studies or expert opinion.

Hence, in epidemiological research, to obtain an accurate estimation of prevalence, misclassification and measurement errors should be considered as part of bias analysis [20]. Bayesian analysis is a useful tool to approach the problem of information bias caused by diagnostic misclassification due to imperfect sensitivity and specificity. Bayesian analysis can incorporate external information by specifying prior distributions on the parameters, such as those obtained from eliciting the opinion of experts, and thereby better estimate disease prevalence and the sensitivity and specificity of diagnostic tests when the true disease state is unknown. Most often, prior knowledge on sensitivity and specificity is incorporated, even though experts often do not have a strong opinion on these test characteristics. For that reason, prior information on conditional probabilities is used [21].

Beyond the immediate operational consequences, accurate prevalence estimates provide the bedrock for strategic decision‐making at multiple levels. For policymakers, such estimates legitimize public expenditure, validate the success of test and slaughter programs, and enable compliance with international trade standards [12, 22]. For field veterinarians, precise estimates provide a critical tool for refining diagnostic decisions, guiding tailored biosecurity interventions, and detecting emerging outbreaks at an early stage [23, 24].

Traditional prevalence estimation methods typically provide single‐point estimates and often require additional corrective measures for errors in the observed data and imperfect diagnostic tests. Machine‐learning algorithms, while designed to optimize predictive accuracy, often fail to show how uncertain those predictions are. However, Bayesian modeling integrates both an estimate and transparent quantification of uncertainty within a single framework, allowing incorporation of prior epidemiological knowledge and diagnostic test characteristics. This makes Bayesian inference particularly important for evidence‐based veterinary studies and policy decision support. Therefore, the present study adopts Bayesian statistical modeling for inference and prevalence estimation rather than relying on machine‐learning methods, since our primary aim is to attain probabilistic estimates of Tp that explicitly account for diagnostic uncertainty, rather than optimizing predictive performance.

This study addresses the gap between observed SICCTT results and the underlying disease reality, using the Holetta Agricultural Research Center dairy farm as a representative case study to demonstrate how a Bayesian analytical framework overcomes the limitations of imperfect tests.

The Holetta dairy farm exemplifies a real operational challenge inherent in bTB control. Despite the implementation of test‐and‐slaughter, its effectiveness remains persistently questioned by farm managers, veterinarians, and policymakers. Conventionally, this question is answered by tracking newly detected cases across successive tests. However, this approach fails when the diagnostic test is imperfect. Without reliable incidence estimates, decision‐makers are rendered blind to the genuine disease status, leaving them unable to determine whether the control strategies are succeeding, failing, or being hindered by specific barriers. The situation at Holetta presents the difficulty of trusting the control program assessments when the metric used for evaluation is itself potentially flawed. The challenge observed at Holetta represents a microcosm of a broader strategic threat.

In the specific context of Holetta farm, the absence of such precision has historically led to premature declarations of successful control on one hand, and the unnecessary culling of valuable animals due to false‐positive results on the other. The fundamental challenge in obtaining reliable insights at Holetta, and in similar settings globally, stems from the inherent limitations of SICCTT with uncertain sensitivity and specificity. It is statistically impossible to solve for Tp, sensitivity, and specificity with certainty using only the results from a single imperfect test.

This study therefore aims to overcome the inherent limitations of imperfect tests by moving beyond biased apparent prevalence (Ap) estimates toward robust, defensible estimates of reality (Tp). By incorporating Bayesian analysis, we transform the raw screening data into high‐quality, intelligence‐grade evidence that empowers veterinarians to refine their clinical decision‐making and equips policymakers to design targeted, economically sustainable interventions for successful bTB control.

The study builds upon the doctoral thesis of Abera [25], which is archived in the Addis Ababa University institutional repository, and provided the foundational dataset and preliminary modeling framework. Building on prior work, this study enhances those findings through Bayesian method refinement, incorporating longitudinal data, and tailoring the outputs into decision‐support tools for veterinarians and policymakers.

This approach provides the Holetta farm and the broader bTB control community with a credible methodology for distinguishing genuine disease trends from artifacts of test performance, thereby enabling evidence‐based refinement.

## 2. Materials and Methods

The study framework was initially developed in Abera’s [25] doctoral thesis. In this document, we adapted and expanded the methodology by providing detailed information about the data source, original sample size, variables used, data quality, and data preprocessing steps applied. These modifications distinguish the present work from the thesis and ensure reproducibility and transparency.

### 2.1. Data Source and Study Design

The study utilized retrospective herd records collected between 2014 and 2019 from a dairy herd maintained at Holetta Agricultural Research Center. The herd was originally established for dairy genetic improvement research and consisted of Boran cattle and crossbred animals. Over the 5‐year period, four consecutive rounds of SICCTT, as described in the OIE Manual of Diagnostic Tests and Vaccines for Terrestrial Animals [26] were carried out to screen for bTB infection in the study herd. In total, 2024 individual screening tests were conducted across the four rounds. Each round included all animals in the herd except the suckler calves and replacement heifers aged ≤ 6 months, which were excluded to avoid confounding due to immature immune responses [25].

The study design was longitudinal, with repeated herd testing and removal of reactors. The time intervals between consecutive test rounds varied (ranging from 345 to 672 days), reflecting irregular implementation of the test and slaughter protocol (Table 1).

**TABLE 1:** Bovine tuberculosis screening test results characterized by test rounds.

Test rounds	Herd size	Test interval (days)	Prevalence (%)		Tuberculin test result		
			Standard	Severe	Negative	Doubtful	Reactors
Test 1	502	—	21.3	27.5	364	31	107
Test 2	521	459	8.4	15.4	441	36	44
Test 3	503	672	24.8	28.2	361	17	125
Test 4	498	345	5.4	9.8	449	22	27

### 2.2. Variables Collected

For each of the four test rounds, a comprehensive set of epidemiological and herd‐level variables was systematically documented. Core analytical variables such as herd size, test intervals, SICCTT results under standard and severe interpretations, and expert‐elicited priors for test characteristics (sensitivity and specificity) formed the basis of a robust epidemiological dataset. This framework allowed for estimation of both apparent and Tp, evaluation of diagnostic test performance, and prediction of herd‐level risks influencing bTB dynamics under imperfect testing conditions. Herd size at the time of testing was the primary epidemiological variable of interest, which provided the denominator for prevalence estimation and allowed herd dynamic assessment.

In addition, test interval (the time interval between consecutive test rounds) was recorded, enabling evaluation of the impact of irregular or prolonged testing schedules on disease transmission. Animals were categorized as negative, doubtful/inconclusive, or reactors (positive). Inconclusive results were treated as negative under standard interpretation, whereas under the severe interpretation they were classified as positive, allowing prevalence comparisons under alternative diagnostic thresholds.

Beyond herd‐level variables, the study incorporated prior information for prevalence and diagnostic test characteristics (sensitivity and specificity). These priors were from previous studies and expert opinions. Specifically, the sensitivity and specificity of SICCTT were parameterized using expert‐elicited values, with Beta priors constructed to reflect realistic uncertainty in diagnostic performance.

#### 2.2.1. Preprocessing and Data Management

Several preprocessing procedures were applied to the dataset prior to analysis to ensure consistency, reproducibility, and methodological rigor. Classification of inconclusive results as either negative or positive was applied to standardize the interpretation, while exclusion criteria were implemented to filter out data that did not meet the study’s requirements and minimize bias. Calves aged 6 months or younger were excluded from all analyses due to their immature immune status, which could compromise test reliability. In addition, animals identified as reactors were not included in subsequent test rounds, thereby preventing misclassification and ensuring that prevalence estimates reflected only the active herd population.

Following classification and exclusion, data were aggregated at the herd level to create a coherent dataset. Ap was calculated as the proportion of reactors relative to the total number of animals tested, providing a standardized measure of disease burden across rounds.

Finally, the data were coded in a format suitable for Bayesian analysis. SICCTT results were further coded into binary outcomes, distinguishing reactors from nonreactors. This coding scheme enabled integration into probabilistic models and facilitated estimation of Tp while accounting for diagnostic uncertainty.

### 2.3. Bayesian Prevalence Estimation

Estimating the Tp in the herd under study, and the true characteristics of the test (i.e., true sensitivity: Se; and true specificity: Sp), was the primary objective of this study. In this study, the definitions of prevalence, sensitivity, and specificity were based on earlier definitions given by Greiner and Gardner [27] and Thrusfield [28]. Ap is defined as the proportion of reactors to the SICCTT in a given test round, and Tp is defined as the proportion of cows infected by *Mycobacteria bovis* in that herd.

**FIGURE 1:**
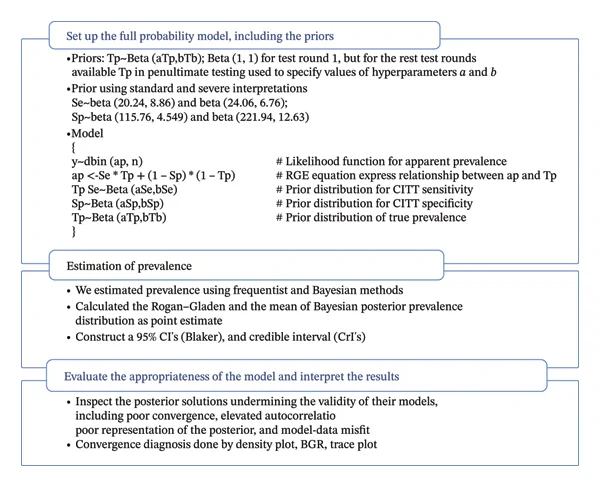
Procedural illustration of study methodology.

In this study, a Bayesian framework was used to estimate the Tp of bTB infection [29]. In keeping with Gelman et al. [30], analysis of this approach was organized around three steps (Figure 1). Briefly, the full probability model including priors was defined in the first step of the analysis, then posterior distributions were estimated in the second step, and the appropriateness of the model was evaluated in the third step. All computations and analyses were programmed using the “rjags” package in the R statistical Software Version 4.1.2 [31], WinBUGS Version 1.4.3 [32], Betabuster (https://www.epi.ucdavis.edu/diagnostictests/betabuster.html), and Bayesian Disease Freedom (BDFree) software, which was downloaded from https://www.epi.ucdavis.edu/diagnostictests/betabuster.html. Supplementary information accompanies this paper; the R codes used as well as all analyzed data from this study are available in the supplemental material.

#### 2.3.1. Setting Priors

The prior distributions of the Tp and test characteristics (sensitivity and specificity) were initially estimated based on previous studies and expert opinion.

Noninformative (uniform) Beta (1, 1) distributions were assigned as the prior for the Tp at first test rounds, according to common practice. However, in the next three successive test rounds, the estimated posterior of the prevalence of bTB infection in the previous test round was used to specify hyperparameter values *a* and *b* for a Beta (*a*, *b*) prior.

The sensitivity and specificity of SICCTT were modeled based on animal health researchers’ opinions conducting the test‐ and slaughter control programs on prospective incidences in a previous study [33]. The mean of their response (expert opinion) values was solicited on SICCTT sensitivity and specificity in the form of a most likely estimate (mode) and a pessimistic estimate (minimum value). The researchers averaged the expected number of reactor animals per infected animal using standard and severe interpretations if SICCTT is applied to some animals of an infected herd. Their results provided 95% confidence that at least 55% and 65% of infected animals (using standard and severe interpretations, respectively) would have positive test results; similar values were reported for specificity. These values were used as inputs in the software Betabuster to parameterize and obtain the shape parameters for the Beta (alpha, beta) prior for sensitivity and specificity.

#### 2.3.2. Model

We set up a model (Figure 1) accounting for uncertainty in the values of Tp, Se, and Sp. It includes the likelihood for each data point and a prior for every parameter of interest to be estimated. Our likelihood functions have three components in this model:

1. The deterministic component expressing the relationship between apparent and Tp given by the Rogan–Gladen estimator equation: Ap = Tp ∗ Se + (1 − Tp) ∗ (1 − Sp)
2. The component linking the response variable “y” to Ap. That is, under binomial sampling, the distribution of the number of animals testing positive is given by the following equation:
(1) yap∼n,ap, and
3. A component to track the priors (Se, Sp) by the model:
(2) Tp∼BetaaTp,bTp,
(3) Se∼BetaaSe, bSe,Sp∼BetaaSp,bSp.
 where aTp, bTp, aSe, bSe, aSp, and bSp are the parameters of the Beta prior distributions for Tp, Se, and Sp, respectively.

Hence, using the above components, the Bayesian model to estimate Tp was mathematically constructed from the conditional distributions as shown in Figure 1. In order to customize the behavior of the model, we encoded our choices for our data model and priors to pass them to the fitting routines in JAGS.

#### 2.3.3. Prevalence Estimation

Here, the data are supplemented with prior information. We assume that Se = Sp = 1 (i.e., ap = Tp). In test round 1, it is assumed that we have little‐known prior testing history and no prior information on disease prevalence in the herd studied. Therefore, a diffuse prior beta (1, 1) for ap was used, which prescribes equal likelihood to every possible value of the prevalence. However, we incorporated posterior penultimate prevalence estimates into the Bayesian analysis to specify values for the hyperparameters *a* and *b* for a beta (*a*, *b*) prior of the consecutive test rounds. Finally, using a binomial distribution:

(4) x∼binn,ap, and

(5) ap∼betaa, b, prior for ap,

the Ap was estimated and, following the Rogen–Gladen formula as detailed above, the posterior distribution of the above model was analytically calculated through the use of Bayesian statistical programs (WinBugs, Jags, Stan) [34, 35].

Furthermore, posterior estimates using informative priors, uninformative priors, and no prior distribution (RGE) were compared in order to explore the influence of the priors over the posteriors in this system. We started with the assumption that Se and Sp are known, fixed values, and then this assumption was relaxed. Our performance metrics to evaluate the influence are the point estimates (median) and the range of the 95% confidence interval (credible interval in the case of the Bayesian method).

#### 2.3.4. Evaluation of the Appropriateness of the Model

Samples were generated by the MCMC algorithm from the posterior distribution to assess convergence, autocorrelation, and representation of the posterior [36, 37]. The convergence of generated Markov chains was checked by density plots, Gelman–Rubin Statistics, and trace plots.

## 3. Result

### 3.1. SICCTT Screening Test Results

The dairy farm conducted a total of 2024 individual screening tests across the four consecutive SICCTT test rounds between 2014 and 2019. Overall, 15% of tests were positive under the standard interpretation and 20.2% under the severe interpretation, representing the overall test positivity across all test rounds. At any given screening test round, prevalence of bTB based on the standard and the severe interpretation ranged from 5.4% to 24.8%, and 9.8%–28.2%, respectively. Comparing test rounds, higher prevalence was recorded in test round 3, while lower prevalence was obtained in test round 4 (Table 1).

### 3.2. Bayesian Analyses

**FIGURE 2:**
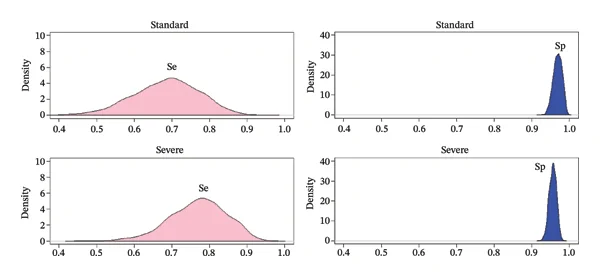
Posterior distribution of sensitivity and specificity of SICCTT using standard and severe interpretation.

The prior distribution of sensitivity of SICCTT using standard and severe interpretations was beta (20.24, 8.86) and beta (24.06, 6.76), while the prior distribution of specificity was beta (115.76, 4.549) and beta (221.94, 12.63), respectively (Table 2). The posterior distributions of Se and Sp are shown in Figure 2. Se estimate for SICCTT was 68.3% using standard interpretation, which was a little bit lower than the prior value (70.1%). However, posterior estimates of Sp using standard and severe interpretations, and Se estimates using severe interpretation were equivalent to those of the prior distributions.

**TABLE 2:** Prior estimates of sensitivity, specificity, and disease prevalence (%) based on expert opinions on the most likely prior (mode), and 95% confidence interval using betabuster analysis.

Parameter	Test	Expert opinion		Beta prior	Prior estimate	
		Mode	95% certain		Median	95% PPI
Sensitivity	SICCTT^a^	0.71	> 0.55	(20.24, 8.86)	70.01	(52.01, 84.56)
	SICCTT^b^	0.80	> 0.65	(24.06, 6.76)	78.67	(62.13, 90.54)

Specificity	SICCTT^a^	0.97	> 0.93	(115.76, 4.55)	96.4	(92.17, 98.84)
	SICCTT^b^	0.95	> 0.92	(221.9, 12.63)	94.74	(91.39, 97.12)

Prevalence	Test round 1			(1, 1)		
	Test round 2^a^	0.1	< 0.276	(2.90, 18.14)	12.65	(2.98, 31.02)
	Test round 2^b^	0.1	< 0.31	(2.44, 13.99)	13.43	(2.65, 35)
	Test round 3^a^	0.27	< 0.41	(10.34, 26.26)	27.86	(15.11,43.65)
	Test round 3^b^	0.31	< 0.42	(18.3, 39.48)	31.44	(20.42, 44.09)
	Test round 4^a^	0.05	< 0.309	(1.488, 10.28)	10.54	(1.0,35.75)
	Test round 4^b^	0.05	< 0.315	(1.47, 9.98)	10.71	(0.99, 36.44)

^a^Standard interpretation.

^b^Severe interpretation.

Estimates (median and 95% Bayesian probability intervals; BPI) of sensitivity, specificity, and Tp for the four testing rounds from the model built are shown in Table 3. In Test round 3, the estimated disease prevalence using standard interpretation criteria was higher than the prior estimate, while all prevalence estimates were lower than the prior estimates in Test round 4 regardless of standard (4.1%) or severe interpretations (6.4%).

**TABLE 3:** Bayesian estimates of disease prevalence (%), sensitivity, and specificity for each test round.

Screening rounds	Parameter	Standard interpretation		Severe interpretation	
		Median	95% PPI	Median	95% PPI
Test 1	Prevalence	27.6	18.5–40.6	31.0	23.1–41.7

Test 2	Prevalence	8.4	3.4–14.2	13.6	8–20

Test 3	Prevalence	30.91	23.3–47.5	31.5	25.1–39.1

Test 4	Prevalence	4.1	0.6–8.6	6.4	1.8–11.8

Overall	Sensitivity	68.3	50.4–83.7	78.1	62.5–90.9
	Specificity	96.8	94.3–98.9	94.5	91.5–97.1

Although true disease freedom is difficult to assess using the model above, we can infer the posterior probability of Tp less than 1%, which is the specified cut‐off to define the herd as free of bTB infection. For test rounds 1, 2, and 3, this probability was 0%, which suggested that there was no chance that the herd was free of *M. bovis* infection. However, in test round 4, there was a probability of 5.8% that the Tp was less than 1%.

### 3.3. Posterior Estimates Using Informative Prior, Uninformative Prior, and With No Prior Distribution (RGE)

Point estimates for Tp using an estimator with informative priors were slightly higher than the median of both RGE and Bayesian estimators with uninformative priors in all test rounds, except Test round 3 (Table 4). The Bayesian estimator with informative priors (unknown Se and Sp) exhibited larger predictive intervals than those with uninformative priors or a frequentist method (RGE). In Test round 3, for instance, the 95% BPI for the posterior estimate assuming unknown but informative priors was (0.233, 0.475), compared to (0.262, 0.372) when using fixed values of Se = 0.70, and Sp = 0.964. The estimate with fixed Se and Sp was much closer to the classical confidence interval (RGE), which was to be expected since both essentially used uninformative priors.

**TABLE 4:** bTB prevalence estimates on subsequent single intradermal comparative cervical tuberculin test.

Test round	Rogan–Gladen point estimate	Bayesian estimate with fixed Se & Sp	Bayesian estimate with informative test prior
Test 1 (*N* = 502)	0.2664 (0.2159, 0.3234)	0.268 (0.215, 0.323)	0.276 (0.185–0.406)
Test 2 (*N* = 521)	0.073 (0.041, 0.114)	0.077 (0.046, 0.114)	0.084 (0.034–0.142)
Test 3 (*N* = 503)	0.3196 (0.2658, 0.379)	0.315 (0.262, 0.372)	0.309 (0.233–0.475)
Test 4 (*N* = 498)	0.0274 (0.0023, 0.0628)	0.033 (0.008, 0.065)	0.041 (0.007, 0.087)

**FIGURE 3:**
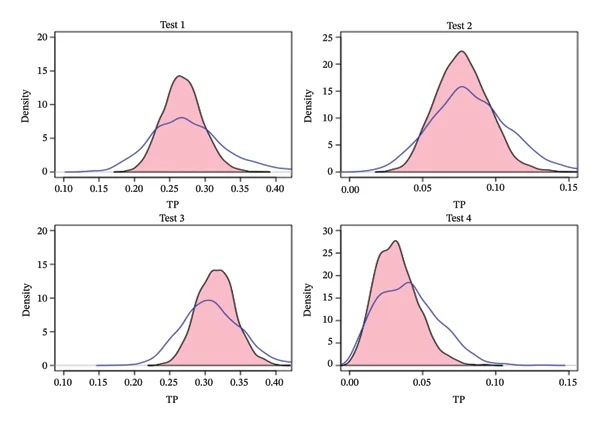
Posterior prevalence distributions, using fixed test sensitivity and specificity (red shaded plot); and unknown sensitivity and specificity (blue line).

Even though coverage is the common performance metric for a confidence (or credible) interval, the precision of these Bayesian estimates was also assessed using density plots. Posterior distributions of Tp estimates with unknown and known Se and Sp were compared (Figure 3). Bayesian estimates with fixed Se and Sp had high‐density posteriors, indicating higher precision than estimates with unknown test Se and Sp. The greater spread of Bayesian estimator posteriors with informative priors represents the loss of precision that results from not knowing the sensitivity and specificity of the test.

### 3.4. Convergence Diagnostics for Markov Chain Monte Carlo (MCMC)

**FIGURE 4:**
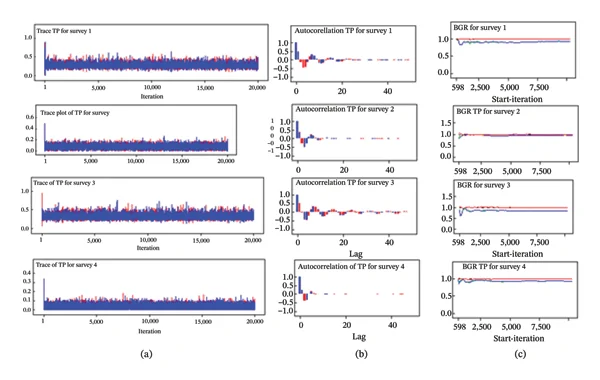
MCMC diagnostic plots illustration.

Convergence of generated Markov chains was verified by trace plots (Figure 4a), autocorrelation function (ACF) plots (Figure 4b), and Gelman–Rubin statistics (Figure 4c). Trace plots of Tp did not indicate nonconvergence of the chains to the stationary distribution and demonstrated proper mixing of the two chains. Sample autocorrelation between the terms of the chains decreased rapidly as a function of their lag, indicating that the quality of these samples was sufficient to provide an accurate approximation of the target distribution. The Brooks Gelman–Rubin convergence diagnosis statistic for Tp estimates also indicated good convergence. Overall, there was no reason to suspect nonconvergence in the current study.

## 4. Discussion

A standard approach for bTB control involves routine field tests, quarantine of infected herds, and removal of positive animals. These measures help to prevent disease spread beyond the herd, while slaughtering infected animals removes the infection from the herd [2]. Nevertheless, achieving this goal is complicated by significant methodological challenges. Inaccurate estimates of disease prevalence and prolonged tuberculin test intervals are likely responsible factors contributing to the persistence of new bTB cases in Holeta dairy farm [38, 39]. Previous studies support the mechanism behind our statement by documenting how imperfect testing complicates bTB control. When bTB tests miss infected animals (false negatives), prolonged intervals give undetected animals more time to transmit infection to susceptible herd mates [17, 40, 41]. All of these negatively impact disease surveillance, control, and eradication programs, and consequently animal trade.

To obtain robust prevalence estimates, our analytical approach incorporated misclassification and measurement errors as part of a comprehensive bias analysis [20]. Lack of knowledge of, or disregard for, test errors (false positives and negatives) can lead to inaccurate or misclassification of diseased states [42].

The present study provides a comprehensive Bayesian analysis of the diagnostic accuracy of the SICCTT for bTB across testing rounds, incorporating expert opinion and previous studies through informative priors. Our findings demonstrate how Bayesian methods can strengthen diagnostic test evaluation in the absence of gold standards, while emphasizing important considerations regarding precision and the added value of incorporating prior information to produce more realistic prevalence estimates.

Our posterior estimates of SICCTT sensitivity (68.3%/78.1%) and specificity (96.8%/94.5%) under standard and severe interpretation align closely with the values reported by Clegg et al. [43] (72.1% sensitivity).

Consistent with the findings of Good [44], our severe interpretation criteria increased sensitivity (78.1% vs. 68.3%) at the expense of slightly reduced specificity (94.5% vs. 96.8%), a common documented trade‐off in diagnostic test research [27].

Notably, our posterior estimates for SICCTT specificity were high (> 94%), supporting its reputation as a specific test for bTB. These findings align with Lahuerta‐Marin et al. [10], who reported > 95% specificity in officially tuberculosis‐free herds. The narrow Bayesian probability intervals for specificity (94.3%–98.9% for standard interpretation) reflect precise estimates from highly informative prior distributions derived from expert opinion.

Previous Ethiopian studies [4–7] have consistently used SICCTT as the primary diagnostic tool and reported Ap estimates without fully accounting for diagnostic uncertainty. While these studies provided valuable epidemiological insights, they did not incorporate Bayesian methods to adjust for misclassification bias. Smith et al. [45] emphasized the importance of frequent herd testing (2–3 months intervals) to limit transmission. However, their study did not apply Bayesian inference to account for uncertainty in prevalence estimates. In contrast, the present study not only confirms the epidemiological importance of testing intervals but also introduces a probabilistic framework that explicitly models diagnostic uncertainty. This dual contribution, a methodological move through Bayesian modeling and contextual application to an endemic herd setting, differentiates the current work from prior studies and provides a foundation for more accurate, evidence‐based, and policy‐relevant prevalence estimation in regions where bTB remains endemic.

Our comparison using different analytical methods (Rogan–Gladen, Bayesian with fixed Se/Sp, and Bayesian with informative priors) yielded varying prevalence estimates. Rogan–Gladen estimate (26.6%) and the Bayesian estimate with fixed Se/Sp (26.8%) were similar for Test round 1, but the Bayesian estimator incorporating Se/Sp uncertainty revealed a different estimate (27.6%) and wider credible intervals (18.5%–40.6%), consistent with prior work of Joseph et al. [46] and Enøe et al. [47] showing that ignoring test accuracy uncertainty causes overprecision.

Expert priors, built with Betabuster, aligned with posteriors. Slight attenuation in standard interpretation sensitivity (posterior median 68.3% vs. prior median 70.0%) shows data modestly adjusted expert priors.

The convergence diagnostics (trace plots, autocorrelation, Gelman–Rubin) indicated satisfactory performance of the MCMC algorithms. Rapid autocorrelation decay and the stabilization of Gelman–Rubin statistics near 1.0 provide evidence that the posterior samples adequately represent the target distributions.

### 4.1. Study Limitations and Future Directions

This study has several limitations. First, the conditional independence assumption across testing rounds embodies a potential source of bias, since repeated sampling of the same cohort is likely to induce correlation. Employing more advanced latent class models that explicitly account for conditional dependence could improve the accuracy of future estimates. Second, the specific attributes of the study population, together with the elicited expert opinions, may constrain the generalizability of our findings to other epidemiological settings.

Additionally, estimates and projections are conditional on the assumptions and dataset available during 2014–2019, which may constrain the robustness of the longitudinal conclusions. Any significant shifts in farm management practices, disease ecology, intervention strategies, or environmental factors beyond this timeframe may alter transmission dynamics and, in turn, impact the predictive performance of the model. To enhance predictive accuracy and policy relevance, periodic model updating using contemporary surveillance data is recommended.

Future research should consider expanding the analytical framework to include multiple diagnostic tests. Evaluating the influence of alternative prior distributions through sensitivity analyses would also be valuable. Most importantly, operationalizing Bayesian prevalence estimates within structured decision‐support tools for bTB control would substantially strengthen both the practical relevance and policy utility of these findings.

## 5. Conclusions

In conclusion, this study demonstrates the value of Bayesian approaches for evaluating diagnostic test performance in the context of bTB surveillance, while highlighting the importance of appropriately accounting for uncertainty in test accuracy parameters. The findings support the continued use of SICCTT as a specific screening test and provide quantitative estimates that can inform evidence‐based policy decisions for bTB control programs.

## Author Contributions

The following authors considerably participated in the study. Berhanu Abera conducted the conceptualization, data curation, formal analysis, investigation, software, methodology design, and preparation of the original draft. Balako Gumi contributed to conceptualization, validation, supervision, and manuscript review and editing. Gobena Ameni provided supervision and contributed to manuscript review and editing. Gezahegne Mamo was involved in conceptualization, validation, supervision, and manuscript review and editing. Rebecca L. Smith contributed to methodology, data curation, validation, visualization, and manuscript review and editing.

## Funding

No funding was received for this manuscript.

## Disclosure

A preprint of this work has been deposited on *SSRN*; https://doi.org/10.2139/ssrn.4529278, allowing early access to the findings while undergoing peer review.

## Conflicts of Interest

The authors declare no conflicts of interest.

## Data Availability

The data that support the findings of this study are available from the corresponding author upon reasonable request.

## References

[1] Vordermeier M. , Ameni G. , Berg S. et al., The Influence of Cattle Breed on Susceptibility to Bovine Tuberculosis in Ethiopia, Comparative Immunology, Microbiology and Infectious Diseases. (2012) 35, no. 3, 227–232, 10.1016/j.cimid.2012.01.003. PMC3339321 22304898

[2] Kao R. R. , Roberts M. G. , and Ryan T. J. , A Model of Bovine Tuberculosis Control in Domesticated Cattle Herds, Proceedings of the Royal Society B: Biological Sciences. (1997) 264, no. 1384, 1069–1076, 10.1098/rspb.1997.0148. PMC1688544 9263472

[3] Nuñez-Garcia J. , Downs S. H. , Parrya J. E. et al., Meta-Analyses of the Sensitivity and Specificity of Ante-Mortem and Post-Mortem Diagnostic Tests for Bovine Tuberculosis in the UK and Ireland, Preventive Veterinary Medicine. (2017) 153, 94–107. 10.1016/j.prevetmed.2017.02.017 28347519

[4] Ameni G. , Aseffa A. , Sirak A. et al., Effect of Skin Testing and Segregation on the Incidence of Bovine tuberculosis, and Molecular Typing of Mycobacterium Bovis in Ethiopia, Veterinary Record. (2007) 161. PMC2292248 18065813

[5] Ameni G. , Vordermeier M. , Aseffa A. , Young D. B. , and Hewinson R. G. , Field Evaluation of the Efficacy of Mycobacterium Bovis Bacillus Calmette-Guerin Against Bovine Tuberculosis in Neonatal Calves in Ethiopia, Clinical and Vaccine Immunology. (2010) 17, no. 10, 1533–1538, 10.1128/CVI.00222-10. PMC2953002 20719984

[6] Guta S. , Regassa A. , Medhin G. , and Ameni G. , Epidemiological Study of Bovine Tuberculosis in Dairy Farms in Addis Ababa, Ethiopia, Preventive Veterinary Medicine. (2014) 114, no. 2, 146–151.

[7] Zewude A. , Berg S. , Engida E. , Hailu E. , Schelling E. , and Ameni G. , Evaluation of the Performance of Bovine Tuberculosis Diagnostic Assays in Dairy Cattle in Ethiopia, BMC Veterinary Research. (2019) 15.

[8] De la Rua-Domenech R. , Goodchild A. T. , Vordermeier H. M. , Hewinson R. G. , Christiansen K. H. , and Clifton-Hadley R. S. , Ante Mortem Diagnosis of Tuberculosis in Cattle: A Review of the Tuberculin Tests, γ-Interferon Assay and Other Ancillary Diagnostic Techniques, Research in Veterinary Science. (2006) 81, 190–210. 10.1016/j.rvsc.2005.11.005 16513150

[9] Lahuerta-Marin A. , McNair J. , Skuce R. et al., Risk Factors for Failure to Detect Bovine Tuberculosis in Cattle From Infected Herds Across Northern Ireland (2004–2010), Research in Veterinary Science. (2016) 107, 233–239, 10.1016/j.rvsc.2016.06.014. 27474001

[10] Lahuerta-Marin A. , Milne M. G. , McNair J. et al., Bayesian Latent Class Estimation of Sensitivity and Specificity Parameters of Diagnostic Tests for Bovine Tuberculosis in Chronically Infected Herds in Northern Ireland, The Veterinary Journal. (2018) 238, 15–21, 10.1016/j.tvjl.2018.04.019. 30103911

[11] World Health Organization (WHO) , Strengthening TB Surveillance to Accelerate Indonesia’s Path to Elimination, 2025, WHO, Indonesia.

[12] World Organization for Animal Health (WOAH) , Controlling Mammalian Tuberculosis: An Ethiopian Pilot Study, 2025, WOAH.

[13] Hutchings S. C. , Randomised Badger Culling Trial Lacks Evidence for Proactive Badger Culling Effect on Tuberculosis in Cattle: Comment on Mills Et al. 2024, Parts I & II, bioRxiv. (2024) https://pmc.ncbi.nlm.nih.gov/articles/PMC11429747/.

[14] Editorial , TB Proposals Sinking Badly, Irish Farmers Journal. (2025) https://www.farmersjournal.ie/viewpoints/viewpoints/editorial-tb-proposals-sinking-badly-868304.

[15] Hogan S. , Six Decades of Failure Around Bovine TB, Irish Farmers Journal. (2025) https://www.farmersjournal.ie/more/northernireland/six-decades-of-failure-around-bovine-tb-884826.

[16] Lockhart C. , Tough Decisions Necessary to Eradicate bTB, Carla Lockhart MP. (2025) https://www.carlalockhartmp.co.uk/tough-decisions-necessary-to-eradicate-btb.

[17] More S. J. , Eradication of Bovine tuberculosis in Ireland: Is It a Case of now or Never?, Irish Veterinary Journal. (2024) https://doaj.org/article/8185a7cde5ff499b91e559d7c2831326. 10.1186/s13620-024-00282-z PMC11662779 39707563

[18] Flor M. , Weiß M. , Selhorst T. , Müller-Graf C. , and Greiner M. , Comparison of Bayesian and Frequentist Methods for Prevalence Estimation Under Misclassification, BMC Public Health. (2020) 20, no. 1, 10.1186/s12889-020-09177-4. PMC7370479 32689959

[19] Berkvens D. , Speybroeck N. , Praet N. , Adel A. , and Lesaffre E. , Estimating Disease Prevalence in a Bayesian Framework Using Probabilistic Constraints, Epidemiology. (2006) 17, no. 2, 145–153, 10.1097/01.ede.0000198422.64801.8d. 16477254

[20] Lash T. L. , Fox M. P. , MacLehose R. F. , Maldonado G. , McCandless L. C. , and Greenland S. , Good Practices for Quantitative Bias Analysis, International Journal of Epidemiology. (2014) 43, no. 6, 1969–1985, 10.1093/ije/dyu149. 25080530

[21] Berkvens D. , Speybroeck N. , Praet N. , Adel A. , and Lesaffre E. , Estimating Disease Prevalence in a Bayesian Framework Using Probabilistic Constraints, Epidemiology. (2006) 17, no. 2, 145–153. 10.1097/01.ede.0000198422.64801.8d 16477254

[22] Rankova R. and Balieva G. , Epizootic Situation of Bovine Tuberculosis in Europe in the Period 2013-2023, Scientific Works. Series C. Veterinary Medicine. (2025) 71, no. 2, 47–52.

[23] Palmer M. V. , Kanipe C. R. , Hwang S. et al., Pathogen Detection in Early Phases of Experimental Bovine tuberculosis, Veterinary Sciences. (2024) 11, no. 8, 10.3390/vetsci11080357. PMC11359862 39195811

[24] Quinn D. , Herd Incidence of TB Increasing While Clinical Cases Drop, Irish Examiner. (2024) https://www.irishexaminer.com/farming/arid-41390596.html.

[25] Abera B. , Modelling Bovine tuberculosis Transmission Dynamics and Disease Control Interventions in Selected Dairy Farms of Ethiopia, 2024, Addis Ababa University Electronic Theses and Dissertations, https://etd.aau.edu.et/handle/123456789/4127, Doctoral dissertation.

[26] Oie M. J. , Bovine Tuberculosis. Manual of Diagnostic Tests and Vaccines for Terrestrial Animals, Part 2, Section 2.3, Chapter 2.3.3, 2007, World Organisation for Animal Health, Paris, France.

[27] Greiner M. and Gardner I. A. , Epidemiologic Issues in the Validation of Veterinary Diagnostic Tests, Preventive Veterinary Medicine. (2000) 45, no. 1-2, 3–22, 10.1016/s0167-5877(00)00114-8. 10802331

[28] Thrusfield M. , Veterinary Epidemiology, 2007, Blackwell Science, Oxford, UK.

[29] Taylor J. M. , Overview and Illustration of Bayesian Confirmatory Factor Analysis With Ordinal Indicators, Practical Assessment, Research and Evaluation. (2019) 24.

[30] Gelman A. , Carlin J. B. , Stern H. S. , Dunson D. B. , and Vehtari A. , Bayesian Data Analysis, 2014, CRC Press.

[31] R Core Team, R.D.C. , R: A Language and Environment for Statistical Computing, 2021, R Foundation for Statistical Computing, Vienna, Austria, https://www.r-project.org/.

[32] Spiegelhalter D. , Thomas A. , Best N. , and Lunn D. , WinBUGS 1.4.3, 2008, MRC Biostatistics Unit, https://www.mrc-bsu.cam.ac.uk/.

[33] Emeru B. A. , Werid G. M. , and Abera B. , Evaluation of Comparative Intradermal Tuberculin and Interferon Gamma Release Tests for Prediction of Tuberculous Lesion Development in Slaughtered Cattle, Journal of Veterinary Medicine and Animal Health. (2020) 12, 78–84.

[34] Carpenter B. , Gelman A. , Hoffman M. D. et al., Stan: A Probabilistic Programming Language, Journal of Statistical Software. (2017) 76, no. 1, 1–32, 10.18637/jss.v076.i01. PMC9788645 36568334

[35] Lunn D. J. , Thomas A. , Best N. , and Spiegelhalter D. , WinBUGS-A Bayesian Modelling Framework: Concepts, Structure, and Extensibility, Statistics and Computing. (2000) 10, no. 4, 325–337, 10.1023/a:1008929526011.

[36] Depaoli S. and Van de Schoot R. , Improving Transparency and Replication in Bayesian Statistics: The WAMBS-Checklist, Psychological Methods. (2017) 22, no. 2, 240–261, 10.1037/met0000065. 26690773

[37] Lee S.-Y. , Structural Equation Modeling: A Bayesian Approach, 2007, John Wiley & Sons.

[38] Abera B. , Gumi B. , Ambaw M. , Emiru B. , and Mamo G. , The Incidence of Bovine Tuberculosis in a Dairy Herd Practicing Irregular Skin Test and Slaughter Control Program, Asian Journal of Research in Animal and Veterinary Sciences. (2024) 7, no. 2, 83–92, 10.9734/ajravs/2024/v7i2288.

[39] Lakew M. , Tadesse B. , Srinivasan S. et al., Assessing the Feasibility of Test-and-Cull and Test-and-Segregation Approaches for the Control of High-Prevalence Bovine Tuberculosis in Ethiopian Intensive Dairy Farms, Scientific Reports. (2024) 14, no. 1, 10.1038/s41598-024-64884-x. PMC11192749 38906922

[40] Gomez-Buendia A. , Ortega J. , Diez-Guerrier A. et al., Evaluating the Ability of Non-Tuberculous Mycobacteria to Induce Non-Specific Reactions in Bovine Tuberculosis Diagnostic Tests in Guinea Pigs and Cattle, Veterinary Microbiology. (2024) 298, 10.1016/j.vetmic.2024.110250. 39265280

[41] Islam S. S. , Pateras K. , Kabir S. L. , Kostoulas P. , Ward M. P. , and Rahman A. A. , A Systematic Review and Meta-Analysis of Bovine Tuberculosis Occurrence and Burden in Bangladesh, 1970–2023, Epidemiology and Infection. (2024) 152, 10.1017/s0950268824001328. PMC11502424 39417391

[42] Messam L. L. M. , Branscum A. J. , Collins M. T. , and Gardner I. A. , Frequentist and Bayesian Approaches to Prevalence Estimation Using Examples From Johne’s Disease, Animal Health Research Reviews. (2008) 9, 1–23. 10.1017/S1466252307001314 18346298

[43] Clegg T. A. , Good M. , Duignan A. , Doyle R. , Blake M. , and More S. J. , Longer-Term Risk of Mycobacterium Bovis in Irish Cattle Following an Inconclusive Diagnosis to the Single Intradermal Comparative Tuberculin Test, Preventive Veterinary Medicine. (2011) 100, no. 3-4, 147–154, 10.1016/j.prevetmed.2011.02.015. 21474194

[44] Good M. , The Tuberculin Test and Its Role in the Strategic Management and Eradication of Tuberculosis in Cattle, 2011, Utrecht University.

[45] Smith R. L. , Tauer L. W. , Sanderson M. W. , and Gröhn Y. T. , Minimum Cost to Control Bovine Tuberculosis in Cow–Calf Herds, Preventive Veterinary Medicine. (2014) 115, no. 1-2, 18–28, 10.1016/j.prevetmed.2014.03.014. PMC4076834 24703601

[46] Joseph L. , Gyorkos T. W. , and Coupal L. , Bayesian Estimation of Disease Prevalence and the Parameters of Diagnostic Tests in the Absence of a Gold Standard, American Journal of Epidemiology. (1995) 141, no. 3, 263–272, 10.1093/oxfordjournals.aje.a117428. 7840100

[47] Enøe C. , Georgiadis M. P. , and Johnson W. O. , Estimation of Sensitivity and Specificity of Diagnostic Tests and Disease Prevalence When the True Disease State is Unknown, Preventive Veterinary Medicine. (2000) 45, no. 1-2, 61–81, 10.1016/s0167-5877(00)00117-3. 10802334

